# Kinematics of the Final Approach and Take-Off Phases in World-Class Men and Women Pole Vaulters

**DOI:** 10.3389/fspor.2022.835659

**Published:** 2022-04-06

**Authors:** Brian Hanley, Helen J. Gravestock, Mike Hopkinson, Giorgos P. Paradisis, Stéphane Merlino, Athanassios Bissas

**Affiliations:** ^1^Carnegie School of Sport, Leeds Beckett University, Leeds, United Kingdom; ^2^School of Health Sciences, Birmingham City University, Birmingham, United Kingdom; ^3^School of Physical Education and Sport Science, National and Kapodistrian University of Athens, Athens, Greece; ^4^International Relations and Development Department, World Athletics, Monte Carlo, Monaco; ^5^Athletics Biomechanics, Leeds, United Kingdom; ^6^School of Sport and Exercise, University of Gloucestershire, Gloucester, United Kingdom

**Keywords:** track and field, elite-standard athletes, kinematics, sex-based differences, coaching

## Abstract

The pole vault is a highly technical event where the athletes must successfully convert horizontal velocity during the run-up to vertical velocity at take-off. The aim of this study was to compare the kinematics of men's and women's world-class pole vaulting. Video data were collected of the best clearances by 14 men and 11 women at the 2018 IAAF World Indoor Championships using three high-speed cameras (200 Hz). Running velocity, step lengths, step times, and pole angles were measured during the run-up; during take-off, distance from the plant box, angle and velocity of take-off, and relative positions of the foot and hands were measured. Men achieved greater clearance heights with faster run-ups, faster take-off velocities and higher hand grip positions (all *p* < 0.001), with each of the last three steps longer for men when expressed as absolute values (all *p* < 0.001), but not when expressed relative to stature. There were no differences in run-up pole angles, step times, take-off angle, take-off contact time or time from pole plant to take-off. Women differed in their approach and take-off for characteristics affected by stature and strength, such as fewer run-up steps, shorter take-off distances, and lower grip heights. These lower grips result from a shorter, lighter pole, and this disadvantage was greater than slower run-up velocities. Coaches should therefore note that sex-based differences occur in the pole vault that result from anthropometric differences, but which do not negate the adoption of similar technical models of vaulting.

## Introduction

The pole vault is a track and field event at the Olympic Games, World Athletics Championships and all other major championships. It is also one of the 10 events that comprise the decathlon and, like the high jump, is an event where the athlete who clears the bar at the highest set height wins. At its simplest, the pole vault consists of a run-up where the aim is to generate kinetic energy for the athlete-pole system (Grabner, 2004), transferred to vertical movement *via* a planting motion, which causes the pole to bend and subsequently straighten, assisting the athlete in gaining height. According to Frère et al. (2010), coaches have classically divided the pole vault into seven stages, the first three comprising the run-up, the transition with arm elevation in the last three steps, and the take-off including the pole plant. The other four phases relate to movements made after the athlete has taken off. In this study, we have focused on the second and third stages of the pole vault action to analyze the final preparation and take-off aspects of the motion.

A fast run-up is crucial in successful pole vaulting because all of the subsequent energy required to complete the vault is generated during this approach phase. Indeed, previous research has found that each increase in velocity of 1 m/s led to an increase in jump height of 0.5 m for men and 0.6 m for women (Adamczewski and Perlt, 1997). Unsurprisingly, because of its importance and relative ease of measurement, run-up velocity is one of the most commonly reported variables in studies on the biomechanics of pole vault. Typically, run-up velocity is measured near the end of the run-up, and most often during the transition phase when the last three steps are taken. This is usually over a 5-m distance, either from 11 m before the plant box to 6 m before it (“11–6 m”) for men, or from 10 m before the plant box to 5 m before it (“10–5 m”) for women (Adamczewski and Perlt, 1997; Schade et al., 2007). The velocity of the run-up is of course restricted by the constraints of holding the pole and less so by the need for accuracy during the pole plant (Frère et al., 2010). Although the main aim of the run-up is to arrive at the take-off with the maximum velocity possible (Linthorne and Weetman, 2012), it is worth bearing in mind that a fast run-up is necessary but not sufficient for a successful jump (Angulo-Kinzler et al., 1994).

One important purpose of a fast run-up and take-off velocity is to ensure enough energy is available to bend the pole sufficiently that its extension allows the vaulter to move upward. However, the key variable that allows for higher bar clearances is the position of the upper hand on the pole as this is effectively the base from which the vaulter pushes upward and over the bar (Linthorne, 1994). There is a clear link between run-up velocity, grip height, take-off distance and, ultimately, clearance height (Tidow, 1989). Research has shown that higher grips on the pole are enabled by faster run-ups and that these higher grips also allow for more bending of the pole, with the distance between the upper and lower hands also important in generating the torque required to bend it (Angulo-Kinzler et al., 1994). Higher grips on the pole require the athlete to take-off farther from the plant box, meaning the athlete must adjust their whole run-up (e.g., starting position) to maintain the same step length strategy, or alter step length during the approach. In effect, the vaulter must monitor step length during the transition phase to ensure the pole is at the correct angle at take-off so that horizontal velocity is maintained into the jump, the pole absorbs enough energy to bend sufficiently, and the body's center of mass (CM) take-off height is maximized. Vaulters usually hold the pole upward during most of the run-up, dropping it gradually as they approach the plant box (Tidow, 1989).

The men's pole vault was part of the athletics program in the very first modern Olympic Games in 1896, but women's pole vaulting did not appear at those championships until 2000. As in other athletic events, women's performances are lower than men's, and research on those first women Olympic vaulters showed that women demonstrated a different technique of jumping and interacting with the pole (Schade et al., 2004). However, by the IAAF World Championships in 2005, women had started to vault in a more similar way to men, but there were still some differences (Schade et al., 2007). Although women's run-ups are slower than men's, and are correlated positively with clearance height (Frère et al., 2010), these do not explain sufficiently the sex-based differences in pole vaulting (Schade et al., 2007). Previous research has explored aspects of pole vault performances that differentiate men's and women's techniques, such as by using a single camera to analyze the run-up phase (Panoutsakopoulos et al., 2021). However, no comparisons have been made between world-class men and women regarding the second and third phases of pole vaulting, which comprise the final three steps and the take-off phase, and which are the crucial link between the horizontal running phase and the vertical push-up phase. The aim of this observational study was to analyze kinematic factors in the take-off phase in establishing similarities and differences between world-class men's and women's pole vaulting.

## Materials and Methods

### Research Approval

Data were collected as part of the Birmingham 2018 IAAF World Indoor Championships Biomechanics Research Project (Hanley et al., 2019). The use of those data for this study was approved by the IAAF (since renamed World Athletics), who own and control the data and, locally, the study was reviewed and approved by Carnegie School of Sport Research Ethics Committee (application reference: 61250). The participants provided their written informed consent to participate in this study. The study was conducted in accordance with the recognized ethical standards of the Declaration of Helsinki.

### Participants

Fourteen of the 15 finalists from the men's pole vault event (age: 25 ± 4 years; stature: 1.86 ± 0.05 m; mass: 79 ± 6 kg) and 11 of the 12 finalists from the women's pole vault event (25 ± 3 years; 1.69 ± 0.06 m; 59 ± 5 kg) were analyzed. The men were taller and heavier than the women (both *p* < 0.001, *d* ≥ 3.20). The men's sample included the 2012, 2016, and 2020 Olympic Champions (who also contain the current World Record holder) and the 2011, 2013, 2015, 2017, and 2019 World Champions; the women's sample included the 2016 and 2020 Olympic Champions and the 2015, 2017, and 2019 World Champions. One man was excluded as his stature was not available, and one woman was not included as she failed to clear any height. Athletes' dates of birth and clearance heights were obtained from the open-access World Athletics website (World Athletics, 2021), whereas their statures and masses were obtained from Matthews (2017) and online sources.

### Data Collection

High-speed video data were collected using three Sony PXW-FS5 cameras (200 Hz; shutter speed: 1/1250 s; ISO: 2000-4000; FHD: 1920 × 1080 px). Two camera locations were situated on the home straight (of the 200-m track that surrounded the infield) and the third was located about two-thirds of the way along the track's back straight. A calibration procedure was carried out before and after each competition using a rigid cuboid calibration frame (3.044 m^3^) that comprised 24 control points. The frame was positioned in two specific, predefined locations along the runway to ensure an accurate definition of a volume beginning a distance from the plant box (men: 11 m; women: 10 m). This approach produced a large number of non-coplanar control points per calibrated volume and facilitated the construction of local coordinate systems, which were then combined into a global coordinate system. In addition, a Canon EOS 700D camera (60 Hz; shutter speed: 1/1250 s; ISO: 1600-3600; SHD: 1280 × 720 px) was placed along the home straight to record the entire trial from the start of the runway to take-off to count the number of run-up steps taken.

### Data Analysis

The collected video files were imported into SIMI Motion (version 9.2.2, Simi Reality Motion Systems GmbH, Germany) and manually digitized by a single experienced operator to obtain whole-body spatiotemporal and kinematic data for the take-off. An event synchronization technique (synchronization of four critical instants: right foot initial contact, right foot toe-off, take-off foot initial contact and take-off foot toe-off) was applied to synchronize the coordinates from each camera. All 25 athletes took off from their left foot. Each file was first digitized frame-by-frame and, upon completion, adjustments were made using the points-over-frame method (Bahamonde and Stevens, 2006). The Direct Linear Transformation (DLT) algorithm (Abdel-Aziz et al., 2015) was used to reconstruct the 3D coordinates of each anatomical location. de Leva (1996) body segment parameter models were used to obtain data for the CM. A recursive second-order, low-pass Butterworth digital filter (zero phase-lag) was employed, where the cut-off frequencies were calculated using residual analysis (Winter, 2005) and ranged between 8.2 and 11.7 Hz. Run-up velocity was measured by digitizing the athlete's head as a proxy for the CM (Hanley et al., 2021) and calculated as the mean horizontal velocity over the 3rd last and 2nd last steps (toe-off to ipsilateral toe-off), which was mostly within a distance 11.6 m away from the plant box for men, and 10.5 m for women (Schade et al., 2007).

Several spatiotemporal and kinematic variables were obtained from the digitized files and are defined in Table 1. Take-off was defined as the last instant of ground contact before the foot leaves the runway as part of the vaulting motion, and pole plant was defined as the instant when the pole makes first contact with the plant box (observed visually as the frame after which the pole stopped moving forward and started bending). Pole angle, run-up step lengths (toe-off to contralateral toe-off) and other distances were calculated using the 3D still image measurement tool in SIMI Motion. Because of sex-based differences in stature, the spatial measurements of step length, 3rd last step to pit distance, take-off distance, take-off CM height, position of the upper hand on the pole, height of the upper grip and grip width were computed as absolute values and relative to athlete stature, whereby they were referred to as ratios. Because the position of the upper hand on the pole affects clearance height (Linthorne, 1994), and this length affects how close the athlete must get to the plant box, step lengths and take-off distance have furthermore been expressed relative to athletes' upper hand positions. Upper hand positions were measured during the pole drop before planting, as athletes might not have achieved full arm extension during pole plant and differences exist in hand position (to the ground) between athletes using a free take-off (i.e., no contact with the ground at pole plant) and athletes using a “grounded” take-off (i.e., pole plant while the foot is still in contact with the ground).

**Table 1:** Variables analyzed and their description.

**Variable name**	**Description**
Step length	The toe-off to toe-off distance between successive steps
Step length ratio (SLR)	The ratio of the last step length to the 2nd last step length. Values less than 100% indicate that the last step was shorter
3rd last step to pit distance	The distance between the toe-off at the start of 3rd last step to the end of the plant box
Pole angle	The angle between the pole and the ground, measured at toe-off for the 3rd last step, 2nd last step, last step (angle of carry) and take-off (angle of attack). Negative values indicate that the end of the pole held by the vaulter was lower than the pole tip
Step time	The time duration between successive steps (toe-off to toe-off)
Take-off velocity	The resultant velocity of the CM at the instant of take-off
Take-off distance	The horizontal distance from the plant box to the foot tip at take-off. Negative values indicate that the take-off foot was closer to the plant box than the hand
Take-off angle	The angle between the path of the CM and the horizontal at take-off
Take-off foot position	The horizontal distance between the toe of the take-off leg and the upper hand at the instant of take-off
Take-off CM height	The vertical distance between the runway and the athlete's CM at take-off
Take-off contact time	The duration from initial contact of the take-off foot to take-off
Time from pole plant to take-off	The duration between pole plant and take-off
Position of upper hand on pole	The distance from the bottom of the pole to the center of the upper hand on the pole at take-off
Height of upper grip	The vertical distance from the runway to the center of the upper hand on the pole
Grip width	The distance between upper and lower hands on the pole

### Statistics

Results are reported as means ± one standard deviation (SD). All statistical analyses were undertaken using SPSS Statistics 26 (IBM SPSS, Inc., Chicago, IL). Independent samples t-tests were used to compare differences between men and women for all variables; significance was set at *p* < 0.05 (Field, 2009). Pearson's *r* was used to find associations between measured variables and final performance (successful jump height) within the men and women samples. Additionally, Cohen's *d* (Cohen, 1988) was used as an effect size to determine the magnitude of the differences between men and women and considered to be either trivial (*d* < 0.20), small (0.21–0.60), moderate (0.61–1.20), large (1.21–2.00), very large (2.01–4.00) or nearly perfect (>4.00) (Hopkins et al., 2009). In calculating *d*, the pooled standard deviation (*SD*_pooled_) was calculated using Equation (1):


(1) $$\begin{array}{l} SDpooled=\sqrt{}[\left(n_{1}\;-\;1\right)\;\times \;SD_{1}{}^{2}\\ \;+\left(n_{2}-\;1\right)\;\times \;SD_{2}{}^{2})\;÷\left(n_{1}\;+\;n_{2}\;-\;2\right)] \end{array}$$

where *n* and *SD* were the size and standard deviation for each group, respectively.

## Results

**Figure 1:**
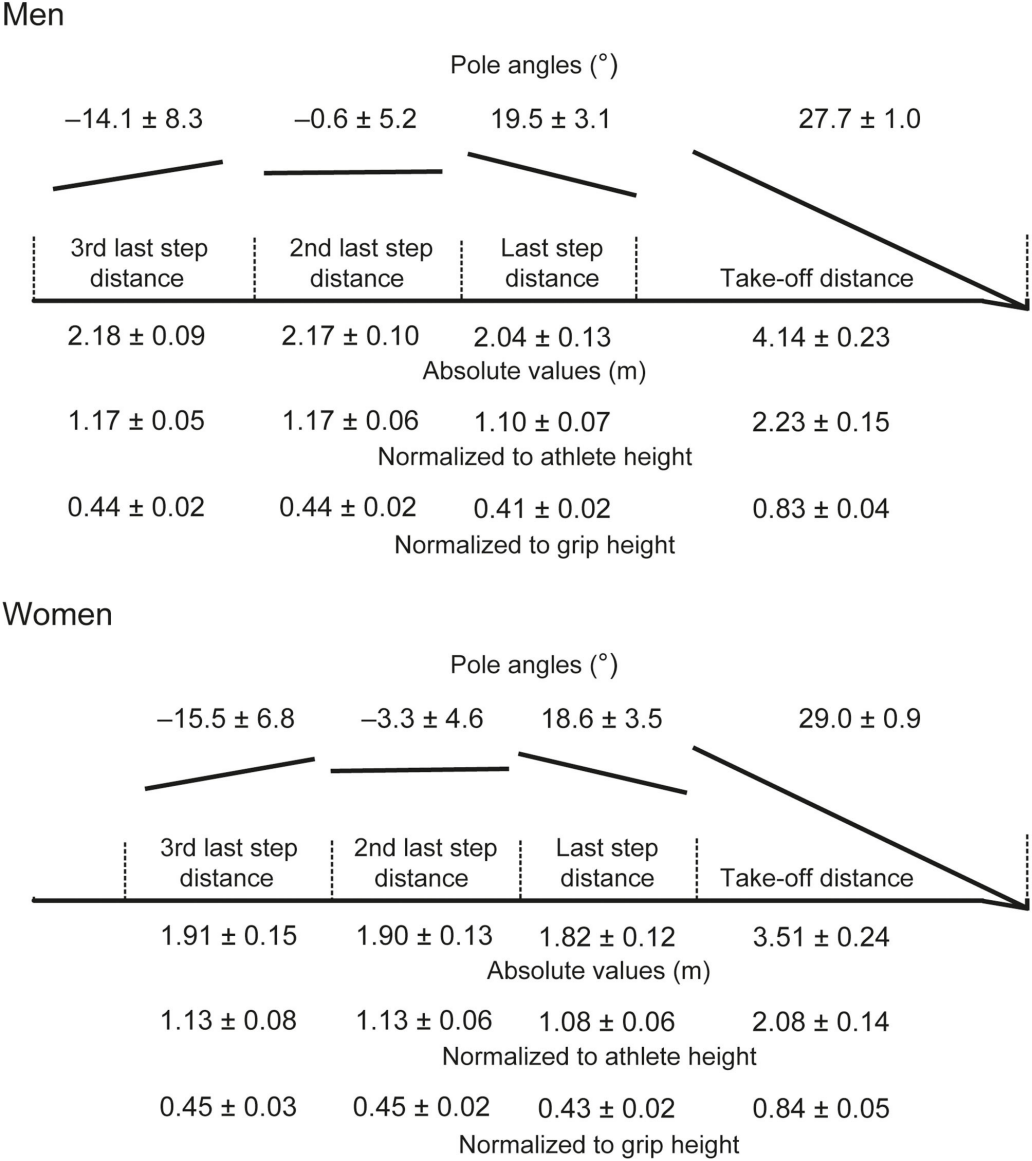
Visual representation of the last three steps and take-off distance, with the angle of the pole during each step included (negative values indicate that the end of the pole held by the vaulter was lower than the pole tip). The plant box is shown as the athletes would approach it running from left to right. The diagram is approximately to scale, with separate diagrams for men and women. The mean values (±SD) are shown as absolute values and, for the distances, as normalized to athlete stature and position of the upper hand on the pole.

The winning height in the men's event was 5.90 m (equivalent to 95.8% of the contemporary men's world record (WR), set by the same athlete), with the last-placed vaulter clearing 5.45 m. In the women's event, the winning height was 4.95 m (97.8% of the women's WR), and the last-placed athlete's highest clearance was 4.50 m. The number of run-up steps was not recorded for one man; the other 13 men averaged 19 steps during their run-up (±2), whereas the women averaged 15 steps (±1). Men were faster than women during the run-up phase (Table 2), with each of the last three steps longer for men when expressed as absolute values (all *p* < 0.001, *d* ≥ 1.78), but not relative to stature (Figure 1). Additionally, there was no difference in the 3rd and 2nd last step lengths when expressed normalized to upper hand position, although the last step was longer in women relative to upper hand position (*p* = 0.016, *d* = 1.05). The mean step length ratio (SLR) values for both sexes (Table 2) show that, on average, the last step was shorter than the 2nd last step; however, two men, including the winner, and two women had longer last steps. Take-off distance was longer for men in absolute values (*p* < 0.001, *d* = 2.71) and relative to stature (*p* = 0.015, *d* = 1.05), but there was no difference when normalized to upper hand position (Figure 1). There were no differences in pole angles (Figure 1) or in step times during the last three steps (Table 2). However, women's pole angles at take-off were greater than men's (Figure 1) (*p* = 0.002, *d* = 1.40).

**Table 2:** Mean ± SD values for run-up spatiotemporal variables.

	**Men**	**Women**	***p***	***d***
Run-up speed (m/s)	9.09 ± 0.23	7.97 ± 0.23	**<0.001**	**4.87**
Step length ratio (SLR) (%)	94.2 ± 6.8	96.0 ± 6.0	0.511	0.27
3^*rd*^ last step to pit distance (m)	10.53 ± 0.37	9.15 ± 0.45	**<0.001**	**3.43**
3^*rd*^ last step time (s)	0.227 ± 0.009	0.232 ± 0.015	0.369	0.40
2^*nd*^ last step time (s)	0.241 ± 0.012	0.239 ± 0.012	0.729	0.14
Last step time (s)	0.202 ± 0.013	0.206 ± 0.014	0.533	0.26

*Between-subject effects (sex-based comparisons) that were significant at p < 0.05 are shown in bold*.

Men had faster resultant take-off velocities than women, with higher velocities at pole plant and take-off (Table 3), although take-off angle, take-off contact time and time from pole plant to take-off were not different. The take-off foot positions were also not different, with most athletes having their take-off foot in front of the upper grip hand (three men, including the winner, and four women, including the silver medalist, had their foot behind their hand). Men had higher take-off CM heights, higher positions of the upper hand on the pole, higher upper grip heights and larger grip widths when expressed as absolute values, but upper grip height and grip width were not different when normalized to stature. There were no correlations between any measured variables and jump height in men but, in women, jump height was correlated with grip height (*r* = 0.74, *p* = 0.010), take-off velocity (*r* = 0.72, *p* = 0.013), horizontal velocity at pole plant (*r* = 0.69, *p* = 0.019), 3rd last step to pit distance (*r* = 0.68, *p* = 0.022), run-up speed (*r* = 0.63, *p* = 0.038), and last step length (*r* = 0.63, *p* = 0.040).

**Table 3:** Mean ± SD values for take-off spatiotemporal variables.

	**Men**	**Women**	***p***	***d***
Take-off velocity (m/s)	8.28 ± 0.35	7.27 ± 0.38	**<0.001**	**2.81**
Take-off CM angle (°)	18.7 ± 1.5	18.8 ± 1.7	0.903	0.05
Take-off foot position (m)	−0.14 ± 0.14	−0.08 ± 0.18	0.347	0.39
Take-off CM height (m)	1.24 ± 0.04	1.09 ± 0.04	**<0.001**	**4.23**
Take-off CM height ratio (%)	66.8 ± 1.2	64.5 ± 1.3	**<0.001**	**1.91**
Take-off contact time (s)	0.115 ± 0.009	0.114 ± 0.006	0.750	0.13
Time from pole plant to take-off (s)	0.064 ± 0.023	0.065 ± 0.028	0.924	0.04
Horizontal velocity at pole plant (m/s)	9.44 ± 0.25	8.15 ± 0.27	**<0.001**	**5.02**
Horizontal velocity at take-off (m/s)	7.84 ± 0.36	6.88 ± 0.39	**<0.001**	**2.61**
Decrease in velocity to take-off (m/s)	−1.60 ± 0.42	−1.28 ± 0.29	**0.042**	**0.86**
Position of upper hand on pole (m)	4.97 ± 0.10	4.20 ± 0.13	**<0.001**	**6.79**
Position of upper hand on pole ratio (%)	267.8 ± 8.3	248.7 ± 3.6	**<0.001**	**0.78**
Height of upper grip (m)	2.25 ± 0.08	2.00 ± 0.08	**<0.001**	**3.21**
Height of upper grip ratio (%)	121.3 ± 3.8	118.3 ± 4.0	0.067	0.78
Grip width (m)	0.68 ± 0.06	0.59 ± 0.05	**0.001**	**1.55**
Grip width ratio (%)	36.7 ± 3.2	35.2 ± 3.9	0.278	0.45

*Between-subject effects (sex-based comparisons) that were significant at p < 0.05 are shown in bold*.

## Discussion

The aim of this observational study was to analyze kinematic factors in the take-off phase in establishing similarities and differences between world-class men's and women's pole vaulting. Because of what previous research has established (Adamczewski and Perlt, 1997), it was unsurprising that men achieved greater clearance heights than women given their faster run-ups, faster take-off velocities and higher hand grip positions. These differences largely result from men's taller statures and greater masses (which we assume includes greater muscle mass) and, indeed, women's smaller masses reduce the amount of energy return available during the pole straightening phase (Schade et al., 2004). However, although take-off velocity has a great effect on the energy absorbed by the pole during the planting motion, the gold and silver medalists in the women's event had faster take-off velocities than the man in last place (who nonetheless achieved a clearance height 0.50 m higher than the best woman), reiterating the concept that a fast run-up is necessary but not sufficient (Angulo-Kinzler et al., 1994), and that run-up velocity is not the crucial factor in differentiating men's and women's pole vault performances (Schade et al., 2007). Tellingly, the last-placed man had a grip height more than 0.70 m higher than any female vaulter, and thus the capability of holding a longer pole higher up is the most obvious explanation of men's better performances (Linthorne, 1994). The women's mean mass was 75% of the mean mass for men, and the lower relative strength of women and the restricting effect of pole mass on velocity (Angulo-Kinzler et al., 1994; Frère et al., 2010) might have influenced their decision to take fewer run-up steps in trying to prevent excessive deceleration toward the end of the run-up.

The absolute spatial differences in men's and women's pole vaulting, including grip height and take-off CM height, suggest that there is a sex-based difference in the model for success in the event (Cassirame et al., 2019). This is unsurprising given how the chosen pole's length and mass must accommodate the athlete's stature and strength. From their study of elite-standard pole vaulters' last eight steps in competition, Panoutsakopoulos et al. (2021) suggested that the main source of sex-based difference in approach velocity was the ability of men to achieve higher power outputs, and it is clear from the present study that women's lower strength has a profound effect on the heights achieved: a shorter, lighter pole must be used; fewer run-up steps are taken; absolute grip width is shorter; and the pole is held lower (even relative to stature), reducing its moment of inertia, but also reducing the amount of bending and, ultimately, the push-off height. Although many of the early successful women pole vaulters were former gymnasts who had excellent technical skills (Bailly et al., 1997), improving power in women vaulters can not only improve performance but also reduce the risk of injury, given the high force magnitudes experienced at pole plant (Schade et al., 2007).

It is clear that men's taller statures and greater strength result in sex-based differences that allow men to vault much higher than women, and these differences do need to be considered by coaches. However, there were enough similarities between the sexes to show that men and women adopt comparable technical models once body size is accounted for, at least during the two phases analyzed. When expressed relative to stature, there were no differences for any of the last three step lengths, the height of the upper grip, or grip width; likewise, when expressed relative to position of the upper hand (i.e., effectively accounting for pole length), there were no differences for the 3rd last and 2nd last step lengths, or take-off distance. The similarities between men and women in many variables were not just limited to normalized values—there were also no differences in step times (and therefore in step frequency) during the run-up; as running velocity is the product of step length and step frequency, this shows clearly that absolute step length differences are the reason for men's faster approach runs. There were also no differences in any other temporal variables, including take-off contact time and time from pole plant to take-off, the latter of which is a key technical skill in vaulting (McGinnis, 1997; Linthorne and Weetman, 2012). Furthermore, the pole drop proceeded in a similar fashion in both groups, as highlighted by the comparable pole angles during the last three steps, and take-off CM angle was virtually identical at just under 19°, similar to pole vaulters from previous research (Linthorne, 2000). Furthermore, take-off foot position and SLR were key spatial measures that did not differ. Notwithstanding that women did have higher pole angles at take-off (albeit by only just over 1°) and lower normalized take-off CM heights, the key skills of pole vaulting differed very little between men and women and these results emphasize the similarities more than the differences. A reduction in the technical gap between the sexes had occurred between the women's event's first Olympic appearance in 2000 and the IAAF World Championships in 2005 (Schade et al., 2007), and we provide novel evidence that this gap has closed further. This is not to say that the aforementioned physical sex-based differences are not important to consider when coaching athletes, but that the fundamental technical model applies equally to men and women.

The biggest advantage of this study is that the results are from the performances of the world's best pole vaulters in competition, providing increased ecological validity because of the maximal intensity of performers and a better understanding of parameters that limit performance (Cassirame et al., 2017). As well as providing for a comparison between elite-standard men and women pole vaulters, the values found show the kinematic characteristics of world-class performances that can be used by coaches. Many of these values were in line with what previous research has found (e.g., Linthorne and Weetman, 2012; Schade and Arampatzis, 2012; Cassirame et al., 2019; Panoutsakopoulos et al., 2021), and supports our understanding of pole vault mechanics by fortifying the current body of research on the event. However, there were also some results that differed from previous studies or, at least, highlighted some exceptions. For instance, it was interesting that although most athletes had their upper grip hand behind the take-off foot, which would be expected to obtain better active energy transmission to the pole (Cassirame et al., 2017), the winner of the men's event had his upper grip in front of the foot. This vaulter also had an SLR greater than 100%, in that his last step was longer than the 2nd last, which is unusual in pole vaulting (Edouard et al., 2019). Hence, in contrast to what Panoutsakopoulos et al. (2021) found in competition, where a sex-based difference was found with four women (but no men) having SLRs above 100%, there was an equal number of two men and two women who achieved this phenomenon in the present study. This suggests that an SLR greater than 100% is not necessarily a technical difference between men and women, but a relatively rare finding that could be specific to those athletes; indeed, it was noteworthy that the winner of the men's competition, and one of the two women, also had SLRs above 100% in a previous global championships (Gravestock et al., 2018). Because our novel study was limited in that we analyzed the best trial for each individual, it was not possible to compare performances at different heights (notwithstanding that some athletes cleared one height only), and the nature of the stadium design precluded the placement of cameras that would allow for full analysis of the bar clearance phase. Because we could not measure individuals' body dimensions, potentially useful values like the Froude number could also not be calculated. Future studies on the biomechanics in world-class competition could explore sex-based differences in other phases of the event, particularly in establishing whether the previously found gap in technique has closed further.

## Conclusions

This novel study compared world-class men and women athletes regarding the second and third phases of pole vaulting, which comprise the final three steps and the take-off phase. Men's longer steps and faster velocities were largely a result of their greater statures, which were not different from women when normalized, and there was no sex-based difference in step times, take-off contact time or time from pole plant to take-off. Several other important elements were identical between men and women, such as the angle of the pole as they lowered it in preparation for planting. However, there were some differences in the take-off phase that could have occurred because of women's smaller masses and lower strength. In particular, women use shorter, lighter poles that naturally result in lower grip heights, shorter take-off distances and less subsequent pole bending. Coaches should therefore note that sex-based differences occur in the pole vault that result from these anthropometric differences, but which do not necessarily negate the adoption of similar technical models of vaulting.

## Data Availability Statement

The datasets presented in this article are not readily available because the data is part of a wider project commissioned by World Athletics. Requests to access the datasets should be directed to BH, b.hanley@leedsbeckett.ac.uk.

## Ethics Statement

The studies involving human participants were reviewed and approved by Carnegie School of Sport Research Ethics Committee, Leeds Beckett University. The patients/participants provided their written informed consent to participate in this study.

## Author Contributions

AB and SM arranged data collection during the World Indoor Championships as Project Director and Project Leader, respectively. BH, HG, MH, and GP performed data collection. BH processed the data and created the figure. All authors conceptualized and designed the study, wrote the manuscript, interpreted the results of the research, edited, critically revised, and approved the final version for submission.

## Funding

The data collection and initial data analysis were supported by funding provided by the IAAF/World Athletics as part of a wider development/education project; however, the nature of the data is purely descriptive and not associated with any governing body, commercial sector, or product. No funding was provided for the writing of this manuscript. The results of the present study do not constitute endorsement by the World Athletics.

## Conflict of Interest

The authors declare that the research was conducted in the absence of any commercial or financial relationships that could be construed as a potential conflict of interest.

## Publisher's Note

All claims expressed in this article are solely those of the authors and do not necessarily represent those of their affiliated organizations, or those of the publisher, the editors and the reviewers. Any product that may be evaluated in this article, or claim that may be made by its manufacturer, is not guaranteed or endorsed by the publisher.
